# Campanacci stage and cortical destruction predict functional outcomes in giant cell tumor of bone

**DOI:** 10.1007/s11845-026-04458-0

**Published:** 2026-05-19

**Authors:** Mehmet Fatih Uzun, Ibrahim Halil Kafadar, Burak Cakar, Fatih Golgelioglu, Anil Aydin, Mehmet Okan Atahan

**Affiliations:** 1 Department of Orthopaedics and Traumatology, Faculty of Medicine, Erciyes University, Kayseri, Türkiye; 2 Department of Orthopaedics and Traumatology, Faculty of Medicine, Bozok University, Yozgat, Türkiye; 3 Department of Orthopaedics and Traumatology, Afyonkarahisar State Hospital, Afyon, Türkiye

**Keywords:** Giant Cell Tumor of Bone, Radiological Parameters, Clinical Predictors, Intralesional Curettage, PMMA, Recurrence, Complications, Functional Outcomes

## Abstract

**Background:**

Although intralesional curettage remains the standard treatment for giant cell tumor of bone (GCTB), the radiological determinants of postoperative functional outcomes remain incompletely defined. This study aimed to evaluate the association between clinicodemographic and radiologic characteristics and functional outcomes, local recurrence, and complications following surgical treatment of GCTB.

**Methods:**

A retrospective analysis was performed on 27 patients treated surgically for GCTB. Demographic variables, Campanacci stage, tumor burden metrics, and MRI features were recorded. Functional outcome was assessed using the Musculoskeletal Tumor Society (MSTS) score. Univariate comparisons were conducted for recurrence and complications. Multivariate linear regression analyses were performed to identify independent predictors of postoperative MSTS scores.

**Results:**

Local recurrence occurred in 3 patients (11.1%). On univariate analysis, Campanacci stage III, soft tissue extension, higher tumor volume, increased tumor-to-bone ratio, and the presence of the paintbrush border sign were significantly associated with recurrence. Postoperative complications were observed in 6 patients (22.2%) and were associated with older age and greater tumor volume. In multivariate linear regression analyses of postoperative functional outcomes, Campanacci stage III and cortical destruction independently predicted lower MSTS scores when evaluated in separate, collinearity-adjusted models (β = − 2.8, *p* = 0.007 and β = − 2.4, *p* = 0.010, respectively).

**Conclusions:**

Functional outcomes following surgical treatment of GCTB are primarily determined by tumor stage and structural integrity rather than demographic factors. Advanced disease and loss of cortical continuity are associated with inferior postoperative function, highlighting the importance of radiologic assessment in surgical planning and patient counseling.

## Introduction

Giant cell tumor of bone (GCTB) is a benign but locally aggressive neoplasm that typically arises in the epimetaphyseal regions of long bones, most frequently around the knee [1, 2]. Although histologically benign, GCTB demonstrates locally destructive growth, causes significant bone loss, and may compromise joint function [1–3]. It constitutes around 5–6% of all primary bone tumors and mainly targets young adults aged 20 to 40 years [4]. The standard treatment remains intralesional curettage together with supportive measures and cavity filling, usually with polymethylmethacrylate (PMMA), which not only provides mechanical stability but also has cytotoxic thermal effects [5]. Despite advances in surgical technique, recurrence rates after curettage remain between 10% and 30%, and postoperative complications such as infection and fracture remain notable challenges [6–8].

To optimize outcomes, numerous prognostic factors have been investigated. Radiological features such as tumor volume, cortical thinning, subchondral distance, and MRI findings (e.g., paintbrush border sign) have been associated with recurrence risk [2, 9]. Similarly, clinical and demographic variables such as patient age, Campanacci stage, and soft tissue extension have been reported as risk factors for recurrence and complications [10–12]. However, most studies to date have primarily focused on recurrence, and functional recovery following treatment has received less attention [13]. Yet for both patients and surgeons, postoperative function is as important as local control, particularly when joint preservation is attempted [14].

Previous investigations suggest that advanced Campanacci stage and cortical destruction may negatively affect function by compromising structural stability and joint congruity [8, 15]. Nevertheless, there is limited evidence directly comparing the predictive value of clinicodemographic versus radiological parameters for functional outcomes. Understanding these relationships is crucial for surgical planning, rehabilitation strategies, and patient counseling.

We hypothesized that functional outcomes, measured by the Musculoskeletal Tumor Society (MSTS) score, are more strongly predicted by Campanacci stage and cortical integrity than by other radiological parameters. The aim of this study was to evaluate both recurrence-related risk factors and functional prognostic factors in patients with GCTB treated with intralesional curettage, thermal cauterization, and PMMA augmentation, with particular emphasis on determinants of postoperative function.

## Methods

### Study design and ethical approval

This retrospective cohort study included patients with histopathologically confirmed GCTB who underwent surgical treatment between January 2013 and June 2022 at our institution. Approval was obtained from the XXX University Clinical Research Ethics Committee (approval number: 2022/755, date: November 9, 2022). Given the retrospective nature, informed consent was waived.

## Patient selection

This retrospective study included patients who were histopathologically diagnosed with GCTB and underwent surgical treatment between January 2013 and June 2022. To achieve uniformity in the cohort, the selection was restricted to patients who received the treatment regimen of intraosseous curettage followed by thermal coagulation and gentamicin-loaded PMMA (polymethylmethacrylate) cement augmentation. Preoperative radiological imaging, which comprised standard radiographs, computed tomography (CT), and magnetic resonance imaging (MRI), was performed for all patients to ensure precise evaluation of tumor size, cortical bone, and subchondral bone involvement. Patients were required to have a minimum clinical and radiological follow-up period of 12 months to evaluate postoperative outcomes and potential recurrence. The analysis excluded patients who had undergone bone grafting, wide en bloc resection, or had incomplete clinical or imaging records. After such criteria were applied, a total of 27 patients were considered suitable for participation in the study.

## Clinical and demographic variables

Data on age, sex, presenting symptoms, tumor location, Campanacci stage, presence of pathological fracture, smoking history, and soft tissue extension were collected. Age was analyzed both as a continuous variable and dichotomized at 40 years, which was selected a priori for clinical interpretability, as GCTB most commonly occurs in young adults aged 20–40 years. Complications were defined as postoperative infection, postoperative fracture, or delayed wound healing.

## Radiological assessment and measurement reliability

All patients underwent anteroposterior (AP) and lateral radiographs of the affected extremity, contrast-enhanced MRI, and non-contrast CT scans. In cases of suspected metastasis, chest CT and posteroanterior (PA) chest radiographs were reviewed.

### Tumor-to-Bone Ratio and Tumor Volume

**Fig. 1:**
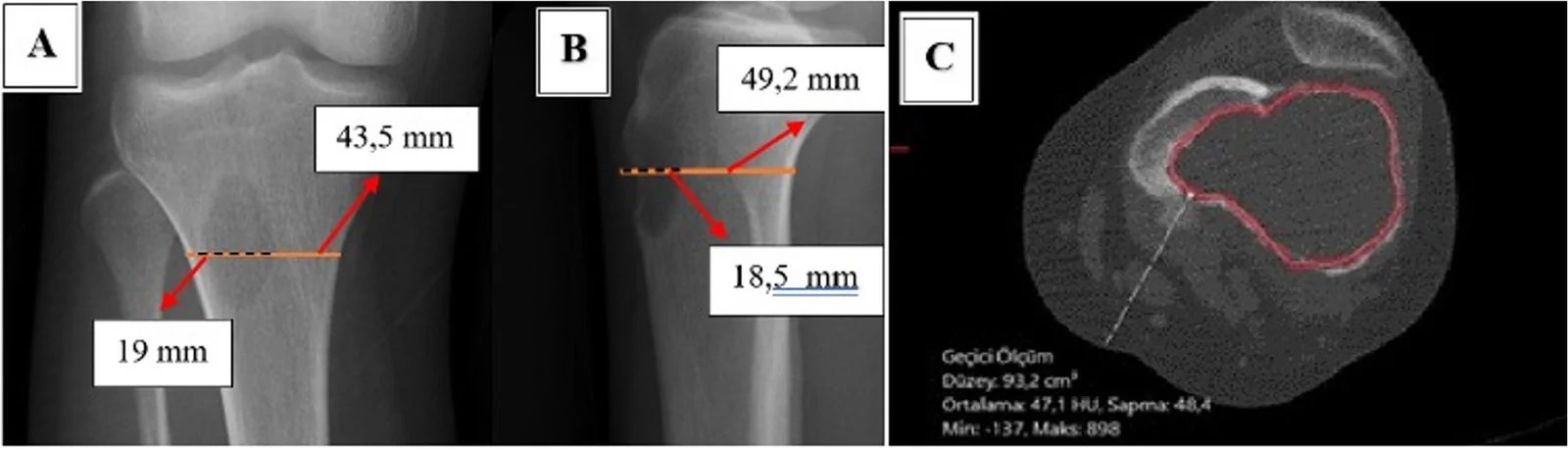
Measurement of tumor-to-bone volume ratio on preoperative CT

On AP and lateral radiographs, the largest tumor diameter was divided by the corresponding bone diameter at the same level to calculate the tumor-to-bone ratio (Fig. 1A–B). Tumor volume was measured on axial CT images using the PACS-integrated volume calculation tool (Sectra UniView, v20.2.9.3362) (Fig. 1C).

### Joint Surface Distance

**Fig. 2:**
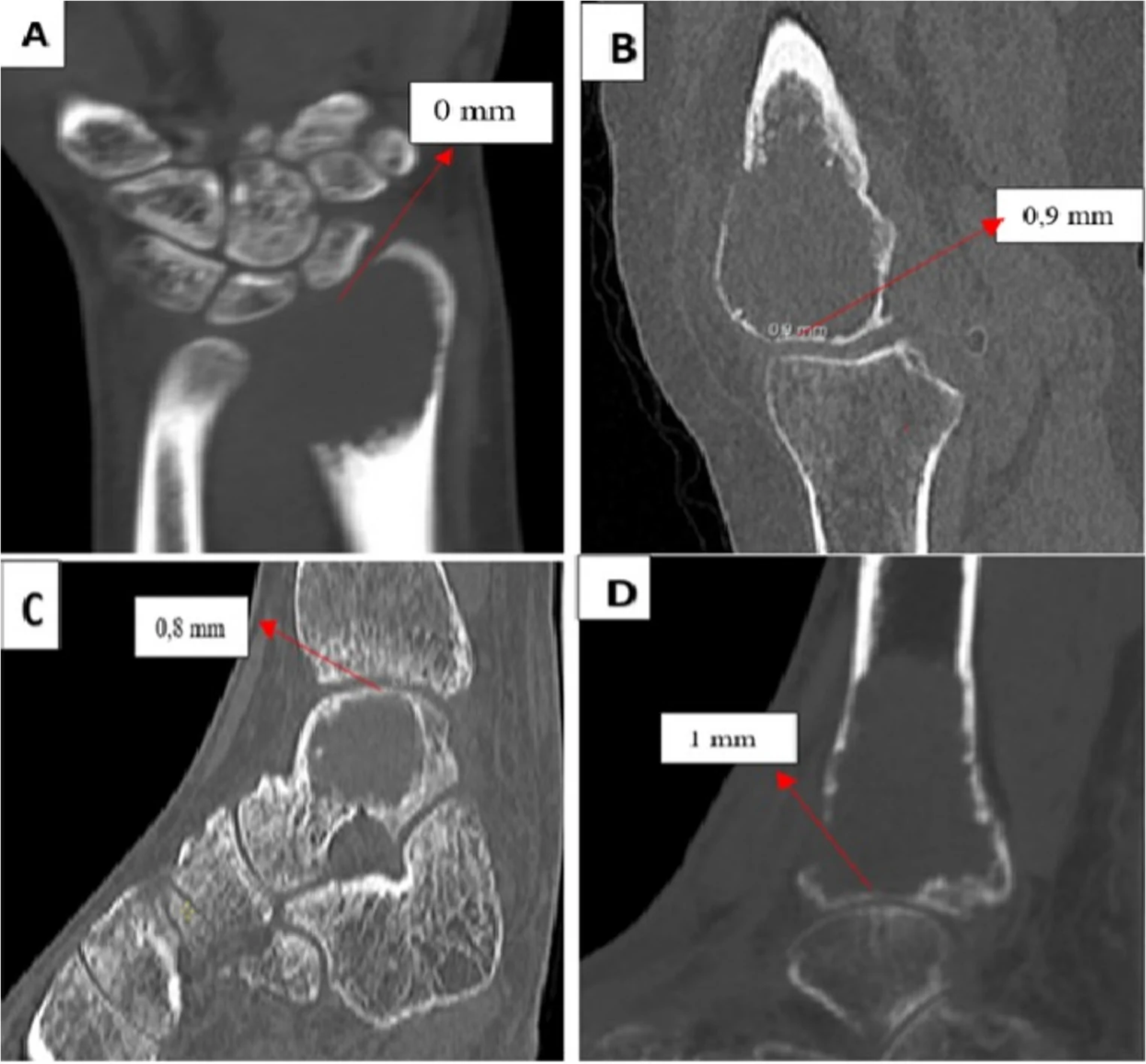
Measurement of the shortest distance between the tumor margin and the articular surface (subchondral distance) on CT scans in various anatomical regions. (A–D) Different cases demonstrating subchondral distances ranging from 0 to 1 mm on coronal and sagittal CT slices

The shortest distance between the tumor margin and the articular surface was measured in coronal and sagittal CT slices and recorded as the subchondral distance (Fig. 2). This measurement was used to assess the extent of articular invasion.

### Thinnest Cortex Thickness

**Fig. 3:**
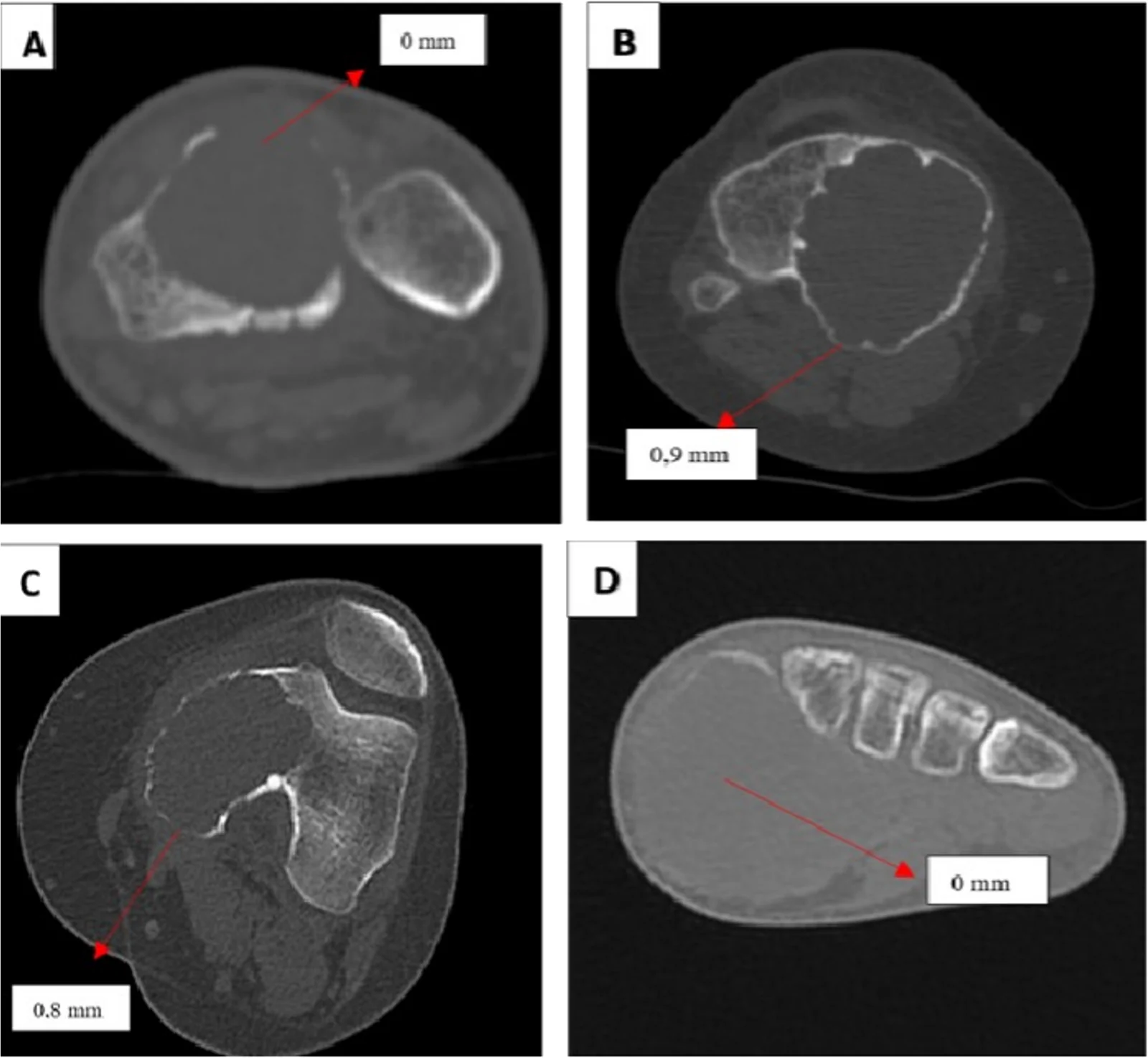
Evaluation of the thinnest cortical bone layer affected by the tumor

In axial CT sections, the thickness of the most thinned cortical area affected by the tumor was measured (Fig. 3). A value of 0 mm indicated complete cortical destruction.

### MRI Findings

**Fig. 4:**
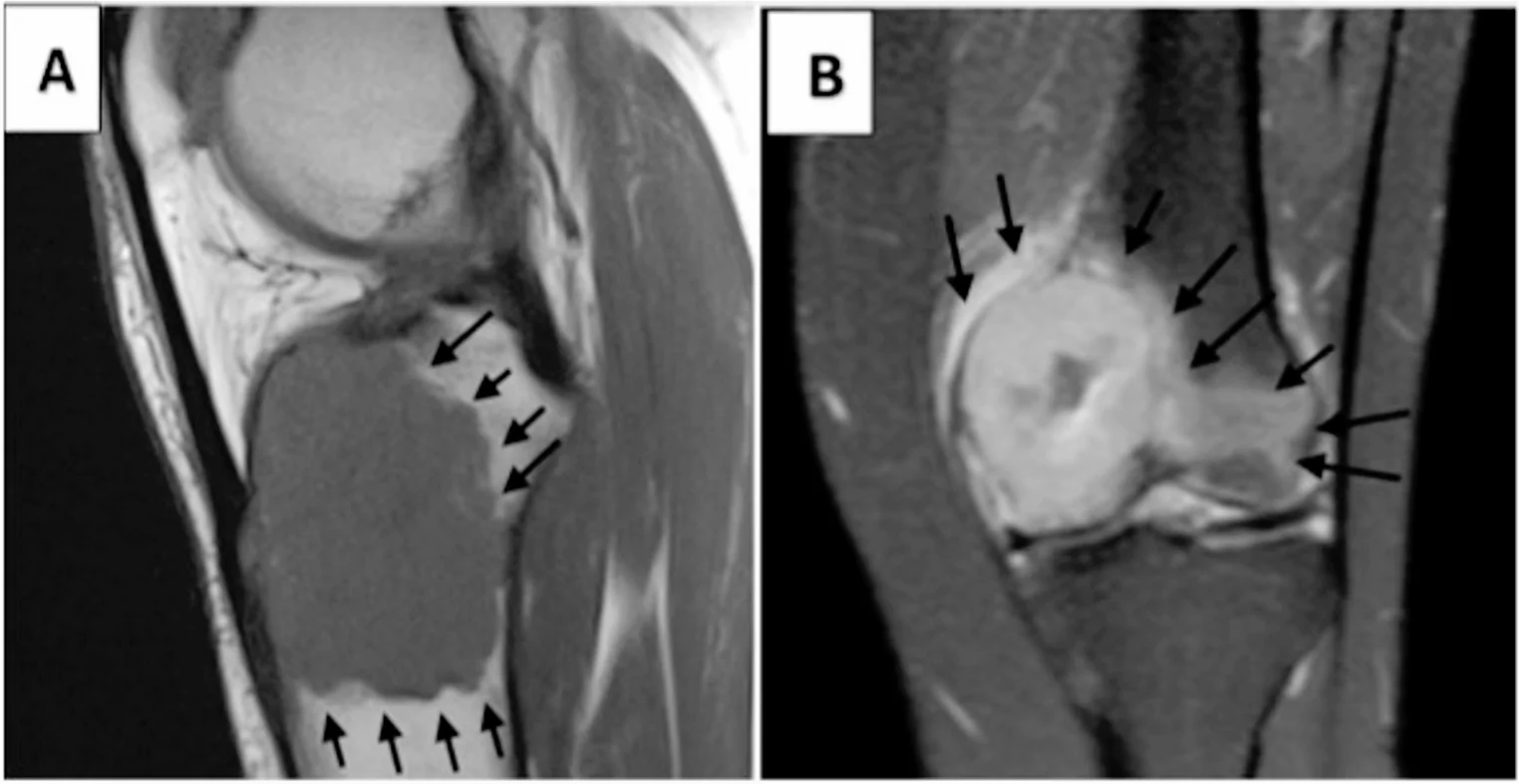
Radiological appearance of the paintbrush border sign and presence of peritumoral edema

Peritumoral edema and the paintbrush border sign were evaluated on T1- and T2-weighted MRI sequences. The paintbrush border sign was defined as irregular signal extensions projecting into the bone. Edema was identified as high signal intensity on T2-weighted images (Fig. 4).

Radiological measurements of cortical thickness and subchondral distance were independently performed by two orthopedic surgeons who were blinded to clinical outcomes. To assess interobserver reliability, measurements were repeated by both observers at two separate time points at least two weeks apart. Intraclass correlation coefficients (ICC) were calculated using a two-way random-effects model with absolute agreement. ICC values were interpreted according to established criteria, with values > 0.75 indicating good reliability and > 0.90 indicating excellent reliability.

## Surgical technique

All patients underwent intralesional curettage performed by the same orthopedic oncology team. After creating a cortical window, thorough curettage and high-speed burring were applied. The cavity was treated with spray-mode thermal cauterization and filled with antibiotic-loaded PMMA cement. Internal fixation was used in cases with severe cortical thinning.

### Follow-up and outcomes

All patients were routinely followed at 3 weeks, 3 months, 6 months, 12 months, and annually thereafter. Magnetic resonance imaging (MRI) was obtained every 6 months during the first two years and subsequently on an annual basis to detect possible local recurrence or postoperative complications. Computed tomography (CT) was obtained selectively when clinically or radiologically indicated, particularly in cases of equivocal MRI findings, detailed cortical assessment, cement–bone interface evaluation, or suspicion of recurrence. The primary oncological outcomes included the occurrence of local recurrence and the incidence of postoperative complications such as infection or pathological fracture. The functional outcome was evaluated using the Musculoskeletal Tumor Society (MSTS) scoring system, which was assessed both preoperatively and at the final available follow-up visit for each patient, in order to reflect the most recent postoperative functional status. Particular attention was given to examining the relationship between Campanacci stage, cortical integrity, and postoperative MSTS scores to explore how radiological aggressiveness and structural compromise influenced long-term functional recovery.

### Statistical analysis

Statistical analyses were performed using IBM SPSS Statistics (IBM Corp., Armonk, NY, USA). Continuous variables were assessed for normality using visual inspection of histograms and the Shapiro–Wilk test. Normally distributed variables are presented as mean ± standard deviation, whereas non-normally distributed variables are reported as median and interquartile range. Categorical variables are presented as frequencies and percentages. Univariate comparisons between the recurrence and no-recurrence groups, as well as between patients with and without postoperative complications, were conducted using Fisher’s exact test for categorical variables due to small cell counts. Continuous variables were compared using the Mann–Whitney U test. A two-sided p-value < 0.05 was considered statistically significant. To identify independent predictors of postoperative functional outcomes, multivariate linear regression analyses were performed with postoperative Musculoskeletal Tumor Society (MSTS) score as the dependent variable. Variable selection for multivariate modeling was based on predefined clinical hypotheses and univariate screening (*p* < 0.10), rather than solely on statistical significance. Age was included in all models as a potential confounder. Given the conceptual and radiological overlap between Campanacci stage III and cortical destruction, these variables were not included in the same regression model to avoid collinearity. Accordingly, two separate multivariate models were constructed: a staging-dominant model incorporating Campanacci stage III, and a structural integrity–dominant model incorporating cortical destruction. Multicollinearity was assessed using variance inflation factors, and no clinically relevant multicollinearity was detected among included variables. Due to the limited number of recurrence and complication events, multivariate modeling was not performed for these outcomes, and results were interpreted descriptively and through univariate analyses. Measurement reliability for radiological parameters was assessed using intraclass correlation coefficients (ICC). Cortical thickness and subchondral distance measurements were independently performed by two observers blinded to clinical outcomes. Interobserver agreement was evaluated using a two-way random-effects model with absolute agreement. ICC values > 0.75 were considered indicative of good reliability, and values > 0.90 indicated excellent reliability.

## Results

### Patient characteristics

A total of 27 patients (14 females, 13 males) with a mean age of 35.2 ± 14.7 years (range, 16–62 years) were included. The most common tumor sites were the proximal tibia (40.7%) and distal femur (25.9%), followed by the distal radius (14.8%), proximal humerus (7.4%), proximal fibula (7.4%), and other locations (3.7%). The median follow-up duration was 29 months (interquartile range [IQR], 14–48 months). At initial presentation, pain was the most common symptom (40.7%), followed by limping (25.9%). Pathological fracture was identified in 3 patients (11.1%), whereas 4 patients (14.8%) were diagnosed incidentally during imaging performed for unrelated reasons. According to the Campanacci classification, 22.2% of tumors were classified as Stage I, 59.3% as Stage II, and 18.5% as Stage III. Soft tissue extension was observed in 5 patients (18.5%), and pulmonary metastasis occurred in 1 patient (3.1%). The mean preoperative Musculoskeletal Tumor Society (MSTS) score was 20.7 ± 4.3 (Table 1).

**Table 1:** Baseline demographic and radiologic characteristics of patients with Giant Cell Tumor of Bone

Variable	Value
Number of patients	27
Age (years), mean ± SD (range)	35.2 ± 14.7 (16–62)
Sex	Female: 14 (51.9%), Male: 13 (48.1%)
Tumor location	Proximal tibia: 11 (40.7%), Distal femur: 7 (25.9%), Distal radius: 4 (14.8%), Proximal humerus: 2 (7.4%), Proximal fibula: 2 (7.4%), Other: 1 (3.7%)
Follow-up duration (months), median (IQR)	29 (14–48)
Presenting symptoms	Pain: 11 (40.7%), Limping: 7 (25.9%), Pathological fracture: 3 (11.1%), Incidental: 4 (14.8%), Other: 2 (7.5%)
Campanacci stage	Stage I: 6 (22.2%), Stage II: 16 (59.3%), Stage III: 5 (18.5%)
Soft tissue extension	5 (18.5%)
Pulmonary metastasis	1 (3.1%)
Preoperative MSTS score (mean ± SD)	20.7 ± 4.3
Tumor volume (cm³), mean ± SD (range)	49.8 ± 44.5 (12–215)
Tumor-to-bone ratio (AP radiograph), mean ± SD	0.64 ± 0.17
Subchondral distance (mm), mean ± SD	4.8 ± 3.2
Cortical thickness (mm), mean ± SD	1.9 ± 0.8
Cortical destruction (> 50% breach)	8 (29.6%)
Paintbrush border sign (MRI)	6 (22.2%)
Peritumoral edema (MRI)	7 (25.9%)

Abbreviations: *MSTS* = Musculoskeletal Tumor Society Score; *AP* = anteroposterior; *IQR* = interquartile range

### Radiological characteristics

Preoperative imaging was available for all patients. The mean tumor volume was 49.8 ± 44.5 cm³ (range, 12–215 cm³), and the mean tumor-to-bone ratio on anteroposterior radiographs was 0.64 ± 0.17. The mean subchondral distance was 4.8 ± 3.2 mm, while cortical thickness at the thinnest point adjacent to the tumor averaged 1.9 ± 0.8 mm. The paintbrush border sign was identified on MRI in 6 patients (22.2%), and peritumoral edema was observed in 7 patients (25.9%). Cortical destruction, defined as loss of > 50% of cortical thickness with radiological cortical breakthrough, was noted in 8 patients (29.6%). These radiological parameters were subsequently evaluated for their associations with recurrence, postoperative complications, and functional outcomes (Table 1). Interobserver reliability analysis demonstrated high agreement for quantitative radiological measurements. Intraclass correlation coefficients (ICC) showed excellent reliability for cortical thickness measurements (ICC = 0.91) and good-to-excellent reliability for subchondral distance measurements (ICC = 0.88), supporting the robustness of imaging-derived parameters used in subsequent analyses.

### Local recurrence

During follow-up, 3 patients (11.1%) developed local recurrence, with a median time to recurrence of 14 months (range, 9–21 months). Univariate comparison between recurrence and no-recurrence groups demonstrated that advanced radiological features were significantly associated with recurrence. Patients with recurrence had a significantly higher mean tumor volume compared with those without recurrence (100.0 ± 78.0 cm³ vs. 41.0 ± 28.0 cm³, *p* = 0.009). Similarly, the tumor-to-bone ratio on anteroposterior radiographs was higher in the recurrence group (0.83 ± 0.13 vs. 0.60 ± 0.16, *p* = 0.03). Campanacci stage III tumors were significantly more frequent among patients with recurrence (66.7% vs. 12.5%, *p* = 0.04), as was the presence of soft tissue extension (66.7% vs. 12.5%, *p* = 0.04). The paintbrush border sign was identified in all recurrent cases and was strongly associated with recurrence on univariate analysis (100% vs. 12.5%, *p* = 0.002). Other variables, including age, sex, smoking status, pathological fracture, subchondral distance, cortical thickness, cortical destruction, and peritumoral edema, did not demonstrate statistically significant associations with recurrence (all *p* > 0.05). Detailed univariate comparisons are presented in Table 2A.

**Table 2:** A. Univariate comparison between recurrence and no-recurrence groups

Variable	Recurrence (*n* = 3)	No recurrence (*n* = 24)	*p*-value
Age (years), mean ± SD	38.0 ± 8.5	34.6 ± 15.1	0.74
Sex (Male/Female), n	2/1	11/13	0.56
Campanacci stage III, n (%)	2 (66.7)	3 (12.5)	0.04*
Soft tissue extension, n (%)	2 (66.7)	3 (12.5)	0.04*
Pathological fracture, n (%)	1 (33.3)	2 (8.3)	0.28
Smoking, n (%)	1 (33.3)	5 (20.8)	0.62
Tumor volume (cm³), mean ± SD	100.0 ± 78.0	41.0 ± 28.0	0.009*
Tumor-to-bone ratio, mean ± SD	0.83 ± 0.13	0.60 ± 0.16	0.03*
Subchondral distance (mm), mean ± SD	3.9 ± 2.6	4.9 ± 3.4	0.55
Cortical thickness (mm), mean ± SD	1.4 ± 0.7	2.0 ± 0.8	0.21
Cortical destruction, n (%)	2 (66.7)	6 (25.0)	0.16
Paintbrush border sign, n (%)	3 (100)	3 (12.5)	0.002*
Peritumoral edema, n (%)	2 (66.7)	5 (20.8)	0.12
B. Univariate comparison between complication and no-complication groups			
Variable	Complication (*n* = 6)	No complication (*n* = 21)	*p*-value
Age (years), mean ± SD	39.2 ± 7.0	28.0 ± 7.1	0.01*
Age ≥ 40 years, n (%)	3 (50.0)	2 (9.5)	0.02*
Sex (Male/Female), n	3/3	10/11	0.95
Campanacci stage III, n (%)	2 (33.3)	3 (14.3)	0.29
Soft tissue extension, n (%)	2 (33.3)	3 (14.3)	0.29
Pathological fracture, n (%)	1 (16.7)	2 (9.5)	0.62
Smoking, n (%)	2 (33.3)	4 (19.0)	0.41
Tumor volume (cm³), mean ± SD	79.5 ± 62.3	42.6 ± 29.7	0.001*
Tumor-to-bone ratio, mean ± SD	0.74 ± 0.19	0.61 ± 0.16	0.06
Subchondral distance (mm), mean ± SD	4.2 ± 2.9	5.0 ± 3.4	0.49
Cortical thickness (mm), mean ± SD	1.6 ± 0.8	2.0 ± 0.8	0.28
Cortical destruction, n (%)	2 (33.3)	6 (28.6)	0.82
Paintbrush border sign, n (%)	2 (33.3)	4 (19.0)	0.42
Peritumoral edema, n (%)	2 (33.3)	5 (23.8)	0.64

Tests: Fisher’s exact (categorical); Mann–Whitney U (continuous). p<0.05 significant

Tests: Fisher’s exact; Mann–Whitney U. p < 0.05 significant

### Postoperative complications

Postoperative complications occurred in 6 patients (22.2%). These included deep infection in 2 patients (7.4%), postoperative fracture in 2 patients (7.4%), and delayed wound healing in 2 patients (7.4%). No cases of implant failure or thromboembolic events were observed. On univariate analysis, patients who developed complications were significantly older than those without complications (39.2 ± 7.0 years vs. 28.0 ± 7.1 years, *p* = 0.01). In addition, age ≥ 40 years was significantly associated with the occurrence of postoperative complications (50.0% vs. 9.5%, *p* = 0.02). Tumor volume was also significantly higher in the complication group compared with patients without complications (79.5 ± 62.3 cm³ vs. 42.6 ± 29.7 cm³, *p* = 0.001). No significant associations were identified between postoperative complications and other clinical or radiological variables, including sex, smoking status, Campanacci stage, cortical thickness, subchondral distance, cortical destruction, paintbrush border sign, or soft tissue extension (all *p* > 0.05). Univariate comparisons are summarized in Table 2B.

### Functional outcomes

The mean preoperative Musculoskeletal Tumor Society (MSTS) score was 20.7 ± 4.3 and improved significantly to 26.9 ± 2.3 at final follow-up (*p* < 0.001). When stratified by Campanacci stage, postoperative MSTS scores were significantly lower in patients with stage III tumors compared with those with stage I–II disease (23.6 ± 3.4 vs. 27.8 ± 2.2, *p* = 0.006). Cortical destruction was also associated with inferior functional outcomes, with lower postoperative MSTS scores observed in patients with cortical breach compared with those without (24.1 ± 3.1 vs. 27.3 ± 2.0, *p* = 0.010). Correlation analyses demonstrated a significant negative association between tumor volume and postoperative MSTS score (*r* = − 0.527, *p* = 0.005), whereas cortical thickness showed a positive correlation with MSTS (*r* = 0.450, *p* = 0.010). Other radiological variables, including subchondral distance, paintbrush border sign, and peritumoral edema, were not significantly associated with postoperative functional outcomes (all *p* > 0.05). Multivariate linear regression analyses were performed to identify independent predictors of postoperative MSTS scores. In the staging-dominant model, Campanacci stage III independently predicted lower MSTS scores (β = − 2.8, *p* = 0.007). In the structural integrity–dominant model, cortical destruction emerged as an independent determinant of inferior functional outcomes (β = − 2.4, *p* = 0.010). Age was included as a potential confounder but did not retain independent significance in either model. Multivariate results are detailed in Table 3.

**Table 3:** Multivariate linear regression models for postoperative MSTS scores Model A – Staging-dominant functional model

Predictor	β coefficient	95% CI	*p*-value
Campanacci stage III	–2.8	–4.7 to − 0.9	0.007*
Age (years)	–0.04	–0.10 to 0.02	0.18
Tumor volume (cm³)	–0.02	–0.04 to − 0.001	0.04*
Tumor-to-bone ratio	–2.1	–4.3 to 0.1	0.06
Subchondral distance (mm)	+ 0.15	–0.05 to 0.36	0.14
Paintbrush border sign	–1.4	–3.2 to 0.4	0.12
Peritumoral edema	–1.1	–2.8 to 0.6	0.20
Model B – Structural integrity–dominant functional model			
Predictor	β coefficient	95% CI	*p*-value
Cortical destruction	–2.4	–4.2 to − 0.6	0.010*
Age (years)	–0.05	–0.11 to 0.01	0.16
Tumor volume (cm³)	–0.02	–0.04 to − 0.003	0.02*
Tumor-to-bone ratio	–1.9	–4.0 to 0.2	0.08
Subchondral distance (mm)	+ 0.18	–0.02 to 0.38	0.08
Paintbrush border sign	–1.2	–3.0 to 0.6	0.18
Peritumoral edema	–0.9	–2.6 to 0.8	0.29

Multivariate linear regression analyses were performed to identify independent predictors of postoperative MSTS scores. Age was included as a potential confounder but did not independently predict functional outcomes in either model. Campanacci stage III and cortical destruction were analyzed in separate models due to conceptual and radiological collinearity

## Discussion

The present study evaluated clinical and radiological predictors of recurrence, complications, and functional outcomes in patients GCTB treated with intralesional curettage and PMMA augmentation. While our findings regarding recurrence risk were consistent with the existing literature—highlighting tumor volume, paintbrush border sign, and soft tissue extension as variables significantly associated with recurrence in univariate analyses—the novel contribution of this study lies in the analysis of functional outcomes. We demonstrated that postoperative function, measured by MSTS scores, was not primarily determined by classical radiological aggressiveness parameters but rather by structural integrity and staging factors. Specifically, Campanacci **Stage** III and cortical destruction were identified as independent predictors of impaired function. Importantly, this study distinguishes predictors of oncological control from determinants of postoperative functional recovery, demonstrating that radiological extent and structural integrity convey different prognostic information.

The demographic characteristics of our cohort generally correspond to prior reports on GCTB, though some deviations were observed. The mean age (35.2 years) and balanced sex ratio are consistent with the large multicenter analysis by Jamshidi et al. [16], which also reported a mean age of 35 years and a slight female predominance. Similarly, the predominance of lesions around the knee—especially the proximal tibia (40.7%) and distal femur (25.9%) —aligns with most series identifying the knee as the most frequent site of involvement [13]. However, unlike those studies, where the distal femur typically outnumbers the proximal tibia, our cohort demonstrated a reversed pattern, possibly reflecting regional variation or referral bias. The Campanacci stage distribution (I 22%, II 59%, III 19%) closely resembles findings from Singh et al. [17], yet is slightly lower in advanced lesions compared with high-volume oncology centers, where Stage III cases reach 25–30% [18]. The rates of soft-tissue extension (18.5%) and pathologic fracture (11.1%) fall within published ranges [10], though the relatively low soft-tissue extension frequency in our series might indicate earlier diagnosis or smaller lesions. The single case of pulmonary metastasis (3.1%) matches the 1–5% incidence summarized in a meta-analysis [19]. Finally, the mean preoperative MSTS score (20.7 ± 4.3) agrees with functional outcome data in knee GCTB cohorts [13], although some reports describe slightly higher baseline function in upper-extremity or low-grade lesions [20]. Overall, our patient distribution and preoperative function are broadly representative of published experience, while subtle differences in site predominance and staging likely reflect institutional and demographic variation.

The mainstay of GCTB management is surgery, including simple or extended intralesional curettage with or without adjuvants, or wide resection [21]. Owing to its periarticular location and high recurrence potential, the optimal approach remains controversial [22]. Recurrence after simple curettage ranges from 30% to 50% [23], whereas extended curettage using a high-speed burr significantly reduces recurrence [24, 25]. Although en bloc resection minimizes recurrence, it requires complex reconstruction and often results in poor functional outcomes [18]. Therefore, local adjuvants are strongly recommended to improve local control [26]. In this study, we observed favourable results with electrocautery and PMMA following extended curettage. Heat application remains an effective adjuvant for reducing recurrence in locally aggressive bone tumors, as electrocautery can destroy tumor cells up to 6 mm beyond the cavity wall [27]. PMMA provided both cytotoxic and mechanical benefits, offering structural support and facilitating early mobilization [28]. Despite potential subchondral thermal damage [29], its role as a reliable adjuvant and early recurrence indicator has been emphasized in large series [30].

Our recurrence rate (11.1%) is consistent with many intralesional curettage series reporting 10–25%. Preoperative CT-based volumetric predictors have shown utility: for example, Zhou et al. [31] demonstrated that CT imaging features (like tumor volume) have prognostic value for recurrence in GCTB. The strong association we found between the paintbrush border sign and recurrence is well supported: He et al. [9] showed that patients with this MRI feature had significantly higher recurrence (71.4% vs. 21.9%) and that it serves as an independent prognostic factor. Parmeggiani et al. [2] also discuss this imaging marker as relevant in risk stratification. Soft tissue extension’s correlation with recurrence in our study aligns with older literature pointing to extraosseous spread as a risk factor. In contrast, we did not observe relationships between recurrence and cortical thickness or subchondral distance, which diverges from some CT-based studies (e.g., Zhou’s CT studies suggested cortical involvement or smaller tumor–articular distances may trend toward higher recurrence) [32]. These inconsistencies likely reflect differences in measurement techniques, surgical margins, or sample sizes.

Our postoperative complication rate (22.2%) is within the higher end of rates reported in the literature, where complication frequencies often vary between 10% and 25%. The finding that older age is associated with increased complication risk resonates with general orthopaedic oncology observations, though it is not always emphasized in GCTB-specific series. Regarding tumor volume, Tsukamoto et al. [8], in a large series of 284 extremity GCTBs, reported postoperative fractures (3.5%) and emphasized that lesion anatomical site, pathological fracture at presentation, and bone grafting use influenced fracture risk, implying that higher volume or structural compromise could predispose to mechanical complications. Moreover, Barnaba et al. [33] describes that across GCT surgical cohorts, non-oncologic complications including infection, mechanical failure, and wound problems are an important aspect of long-term morbidity. However, we did not find associations between complications and radiological parameters like cortical thickness or soft tissue extension, which contrasts with some biomechanical or finite element studies suggesting that cortical weakening or extraosseous spread can compromise structural integrity. Also, in Ebeid et al. [19] on knee GCTs with pathological fractures, complication rates after curettage + cementation were relatively low (10%) despite fracture presence, which may reflect differences in surgical technique, bone quality, or load protocols among cohorts.

Our findings that the mean MSTS score improved significantly from 20.7 preoperatively to 26.9 postoperatively (*p* < 0.001) underscore the meaningful functional recovery achievable after surgical intervention. This aligns with reports like Yadav et al. [13] in knee GCTB, where extended curettage/cementation led to high MSTS scores in follow-up (mean ~ 28–29) across tumor grades. Moreover, we observed that **Stage** III (Campanacci III) lesions had significantly lower postoperative MSTS (23.6) compared to **Stage** I–II cases (27.8), supporting the notion that more aggressive radiological staging may limit functional gains. This is consistent with the study by Saputra et al. [20], where they suggest that more aggressive tumors yield lower functional scores. While we observed cortical destruction as an independent predictor of lower MSTS scores, Kumar et al. [34] focused on cortical involvement as a determinant of local recurrence and reported preserved functional outcomes with appropriate mechanical stabilization. These findings indicate that cortical integrity may have different implications for oncological control and functional recovery. The association between cortical destruction and inferior postoperative MSTS scores may be explained by the biomechanical and surgical consequences of cortical compromise. The cortex serves as a primary structural pillar of the metaphyseal bone, contributing to load transmission, joint stability, and resistance to deformation. Extensive cortical loss may lead to altered load distribution across the adjacent joint, increased micromotion at the reconstruction site, and a greater need for soft-tissue dissection or mechanical stabilization during surgery. These factors can adversely affect postoperative muscle function, joint congruity, and overall limb mechanics, thereby limiting functional recovery despite adequate oncological control.The negative correlation between tumor volume and MSTS (*r* = − 0.527) supports the idea that bulkier tumors limit postoperative function, a relationship hinted in prior imaging-based evaluative systems in GCT [35]. The observed positive correlation of cortical thickness with MSTS (*r* = 0.450) is intuitively plausible but less frequently reported; most literature focuses on cortical breach rather than thickness. The lack of significant associations of subchondral distance, paintbrush border sign, and peritumoral edema with MSTS diverges from some studies that propose MRI invasive signs may reflect more aggressive infiltration — this discrepancy might stem from sample size, measurement variability, or anatomical site differences. Although a shorter subchondral distance is theoretically expected to compromise joint stability and predispose to secondary degenerative changes, this parameter did not show a significant association with postoperative MSTS scores in the present cohort. Several factors may account for this finding. First, functional assessment in this study reflects short- to mid-term outcomes, whereas the detrimental effects of subchondral proximity—such as progressive cartilage degeneration or post-cement arthropathy—may manifest over longer follow-up periods. Second, surgical reconstruction strategies, including subchondral bone grafting and careful cement placement, may have mitigated the mechanical and thermal impact of PMMA on the articular surface, thereby preserving early functional outcomes. Finally, the limited sample size may have reduced statistical power to detect a modest functional effect, raising the possibility of a type II error.

In summary, our multivariate model delineated distinct predictors across outcome domains. Local recurrence was chiefly associated with radiological aggressiveness—specifically tumor volume and the paintbrush border sign, consistent with Zhou et al.’s CT-based recurrence prediction study [31] and He et al.’s MRI paintbrush border pilot work [9]. In contrast, complications were more closely linked to patient age and tumor size, aligning with functional outcome studies that show larger lesions carry higher surgical risk as seen in the Yadav et al. series [13]. Finally, functional outcomes were predominantly determined by structural integrity and staging factors: our finding that Campanacci **Stage** III lesions yielded lower postoperative MSTS is compatible with observations reported by Johan et al. [36], while the negative correlation between tumor volume and MSTS echoes trends in the Ebeid et al. knee GCT series [19]. These findings underscore that recurrence, complications, and function are governed by overlapping but distinct prognostic axes, and that surgical planning for GCTB must balance oncologic control with preservation of structural and functional potential.

### Clinical implications

The present findings highlight the importance of individualized risk assessment in the management of GCTB. Radiological aggressiveness (tumor volume, paintbrush border sign, and soft-tissue extension) should alert surgeons to an increased likelihood of recurrence, whereas patient age and lesion size may guide perioperative precautions to prevent complications. In contrast, preservation of cortical integrity and avoidance of excessive subchondral violation appear essential for optimal postoperative function. Integrating both oncologic and functional prognostic factors into surgical planning may enhance local control while maintaining long-term joint performance.

### Strengths and limitations

This study provides a comprehensive evaluation of both oncologic and functional outcomes in patients with giant cell tumor of bone treated using a uniform surgical protocol combining curettage, electrocautery, and PMMA augmentation. Quantitative assessment of radiological variables such as tumor volume, cortical integrity, and MRI features allowed an objective correlation with postoperative function. Furthermore, integration of MSTS scoring enabled a meaningful link between radiological aggressiveness and patient-reported functional recovery, an aspect rarely addressed in prior studies. Nevertheless, the retrospective single-center design and modest sample size may limit generalizability. The median follow-up of 29 months, while comparable to that of most recent series, may still underestimate very late recurrences. In addition, the very small number of recurrence events (*n* = 3) limited statistical power for oncologic endpoint modeling; therefore, multivariable analysis for recurrence and complications was not performed and these results should be interpreted descriptively. Reconstruction strategy (cement alone versus subchondral bone grafting/sandwich approaches) and the use of internal fixation were not captured as separate analytic variables; these individualized surgical decisions may modify postoperative biomechanics, mitigate cement-related effects on subchondral bone, and influence functional recovery, particularly in cases with cortical compromise or joint proximity. Pathological fracture at presentation and baseline functional reserve may also act as potential confounders; although baseline MSTS scores were available, a standardized assessment of pre-injury/pre-symptom function was not uniformly recorded. Minor interobserver variability in radiological measurements also remains a potential limitation despite standardized evaluation criteria. To strengthen the reliability of imaging-derived variables, we formally evaluated interobserver agreement for key radiological measurements (including cortical thickness and subchondral distance) using intraclass correlation coefficients (ICC), demonstrating high agreement; however, some degree of measurement error is unavoidable in retrospective imaging-based analyses. Finally, given the modest cohort size, non-significant findings for certain radiological parameters may reflect limited power (type II error) rather than true absence of association.

## Conclusion

Radiological aggressiveness and structural integrity represent distinct yet complementary predictors in the management of giant cell tumor of bone. Tumor volume, paintbrush border sign, and soft-tissue extension primarily determine recurrence risk, whereas patient age and lesion size influence complication rates. In contrast, functional outcomes are mainly governed by cortical preservation and staging severity. Recognizing these separate prognostic domains can help surgeons tailor treatment strategies that balance oncologic safety with optimal postoperative function.

## Data Availability

The datasets used and analyzed during the current study are available from the corresponding author upon reasonable request.
